# Issues around the Prescription of Half Tablets in Northern Switzerland: The Irrational Case of Quetiapine

**DOI:** 10.1155/2015/602021

**Published:** 2015-10-11

**Authors:** Samuel S. Allemann, Delia Bornand, Balthasar Hug, Kurt E. Hersberger, Isabelle Arnet

**Affiliations:** ^1^Pharmaceutical Care Research Group, Pharmaceutical Sciences, University of Basel, Klingelbergstrasse 50, 4056 Basel, Switzerland; ^2^Hospital Pharmacy, University Hospital Basel, Spitalstrasse 26, 4031 Basel, Switzerland; ^3^Internal Medicine, University Hospital Basel, Spitalstrasse 21, 4031 Basel, Switzerland

## Abstract

*Background*. Prescription of fragmented tablets is useful for individualisation of dose but includes several drawbacks. Although without score lines, the antipsychotic drug quetiapine was in 2011 the most often prescribed 1/2 tablet in discharge prescriptions at the University Hospital in Basel (USB, 671 beds). We aimed at analysing the prescription patterns of split tablets in general and of quetiapine in particular in Switzerland. *Methods*. All orders of community pharmacies for unit-of-use soft pouch blisters placed at Medifilm AG, the leader company in Switzerland for repackaging into pouch blisters, were analysed. *Results*. Out of 4,784,999 tablets that were repacked in 2012 in unit-of-use pouch blisters, 8.5% were fragmented, mostly in half (87.6%), and were predominantly psycholeptics (pipamperone 15.8%). Prescription of half quetiapine appears to be a Basel specificity (highest rates of fragments and half quetiapine). *Conclusions*. Prescription of fragmented tablet is frequent. It represents a safety issue for the patient, and a pharmaceutical care issue for the pharmacist. In ambulatory care, the patient's cognitive and physical capacities must be clarified, suitability of the splitting of the tablet must be checked, appropriate aids must be offered, like a pill-splitting device in order to improve accuracy, and safe use of the drug must be ensured.

## 1. Introduction

Previous studies showed that fragmenting concerns every fourth tablet in ambulatory setting [1, 2] predominantly because of dose adjustment, swallowing difficulties, or costs [3–5]. However, some drawbacks exist such as breaking difficulties, breaking in unequal parts, and loss of mass [5]. Further, changing the dosage form may degrade the active substance at the fractured surface and thus alter its absorption characteristics. The site of action may not be reached, which may be clinically relevant, especially for substances with narrow therapeutic index [6]. The keeping of the halves may be difficult because of problems of stability and of identification. Further, controlled release forms are unsuitable for splitting, since their destruction can lead to dose-dumping and dose-dependent side effects by altering the liberation kinetics of the substance. Finally, substances with irritating or toxic properties, especially the CMR substances (carcinogen, mutagen, or toxic for reproduction), should be split only with protective measures (e.g., gloves and masks) [7].

The European regulatory authorities evaluated splitting tablets into segments [8]. This apparently simple operation bears a potential for dosage error that increases if the tablets are not scored. In view of the many exceptions where splitting is not allowed (enteric coated tablets, layered tablets, and many modified release dosage forms), the authorities concluded that manufacturers should provide information on the issues surrounding cutting tablets into smaller segments. In USA, the FDA, the American Medical Association, and other medical organizations consider tablet splitting as a risky practice and advise against it unless it is specified in the drug's labeling [9]. The analysis of electronic medication regimens from 54 wards of a large university hospital in Germany showed that 12.5% of all drugs were prescribed in split form [10]. Splitting was inappropriate for 2.7% of all drugs, mainly because of the absence of a score line. A retrospective study performed at the University Hospital Basel in Switzerland showed similar results [11]. Of the 36,751 electronic prescriptions delivered in 2011 at discharge, 3,724 (10.1%) contained the mention “1/2” and concerned 4,888 single tablets. Of those 1/2 tablets, 16.4% were wrongly prescribed, predominantly due to inexistent score lines. Quetiapine (Seroquel, Sequase 25 mg), a tablet with no score line, was the drug most often wrongly prescribed as half tablet.

Quetiapine is an atypical or second-generation antipsychotic agent similar in structure to clozapine and exhibits strong antagonism of 5HT_2_ receptors and weak antagonism of D_2_ receptors [12]. It is approved for the treatment of schizophrenia and bipolar disorders [13] and is widely used mainly because it does not induce agranulocytosis [14] and thus does not require blood monitoring. Its substantial advantage is further a favourable profile of acute extrapyramidal side effects that occur in very rare cases [15]. Off-label use, that is, unlabeled or unapproved use, is common in conditions such as agitation, anxiety, dementia, obsessive-compulsive disorders, psychosis [16], and delirium [17, 18]. Because of many inconclusive study results, evidence is limited. A meta-analysis of seven randomized controlled studies with 3,257 participants evaluated the effects of quetiapine for anxiety disorders at doses ranging between 25 and 400 mg/day [19]. Monotherapy with quetiapine was better than placebo in reducing symptoms of generalized anxiety disorder and was equivalent to antidepressants in improving depressive symptoms. In all studies, more subjects in the quetiapine group left the trials early due to adverse events (gained weight and sedation). The additional use of quetiapine at doses between 25 and 600 mg/d was established in a further meta-analysis only in the treatment of generalized anxiety disorder [16]. The small clinical studies mostly started doses at 25 mg/day [20–22]. We were able to find low-dose quetiapine at 12.5 mg only in one Italian study for the initiation of treatment in 41 patients with dementia and concomitant psychotic disorders [23] and in one Spanish study with 7 Parkinson's patients, where low-dose quetiapine was effective on psychotic symptoms, sleep disturbances, and stress of the caregivers [24].

Building up on the local observation of 2012, we aimed at analysing the general prescription patterns of split tablets in Switzerland. Thus, the questions of interest are as follows.* “What is the prevalence of split tablets in Switzerland? Is the wrong prescription of half quetiapine tablets restricted to a local habit in Basel?” *Further, we aimed at evaluating the consequences of split tablets for community pharmacies, patients, and patient care organisations and discussing some recommendations for daily practice.

## 2. Material and Methods

We obtained all orders placed by Swiss community pharmacies at Medifilm AG, the leader company in Switzerland in the repackaging of medication into unit-of-use soft pouch blisters, located in the industrial area of Oensingen (canton Solothurn) [25]. Community pharmacists can order rolls of single pouches containing various medications to be taken at one time, mainly for long-term institutionalized patients. Segments of tablets can be ordered without restriction. Orders are submitted to quality assurance checks. When split tablets are required and corresponding lower dosage strength is available as single tablet on the market, an exchange takes place. If no lower dosage strength is available and the formulation of the tablet is conventional (i.e., no enteric coat and no modified release), the tablet is fragmented with an automatic pill-splitter. According to the Summary of Product Characteristics [13], quetiapine tablet is a round, 6 mm in diameter, film-coated tablet without score line. Since its formulation is without functional coating, the splitting of the lowest strength of quetiapine tablet (Seroquel 25 mg original brand and Sequase 25 mg generic brand approved since 09/2011) is performed.

Presence of a score line and suitability for splitting of tablets were obtained from the Swiss Summary of Products Characteristics [13]. Archive files were retrieved from the open drug database http://ch.oddb.org/.

## 3. Statistics

We used the SPSS statistical package version 21.0 (SPSS Inc., Chicago, IL, USA) for data description and the R system for computation and graphics (v3.1.3, R Core Team (2015); R: a language and environment for statistical computing; R Foundation for Statistical Computing, Vienna, Austria, http://www.R-project.org/). Additional graphics were created with Power Map Preview for Excel 2013 (Microsoft Excel [computer software], Microsoft, 2013, Redmond, Washington, USA).

## 4. Results

**Figure 1:**
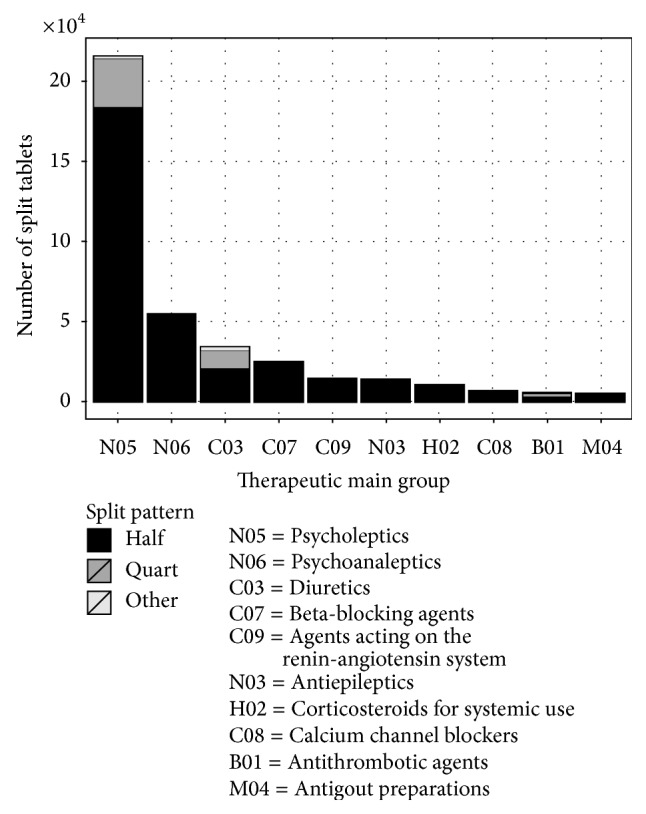
Distribution of the ten most often split tablets sorted by ATC therapeutic main group (*N* = 406,956).

Between January 1 and December 31, 2012, a total of 4,784,999 tablets were packed in unit-of-use soft pouch blisters by Medifilm. Of these, a total of 406,956 (8.5%) were fragments of tablets that had been ordered by 29 community pharmacies for 1,321 patients residing in 53 retirement homes in Northern Switzerland. The homes have used in 2012 between 14 and 48,300 fragmented tablets (Table 1). The patients were in average 81.5 ± 14.7 years old (median: 86; range: 7–105) and obtained in average 1.7 fragments (median: 1; range: 1–8). A total of 577 (43.7%) patients received two or more fragments of tablets (Table 2). The majority of the fragments were halves (356,339; 87.6%) and quarters (45,375; 11.1%) and marginally thirds, two-thirds, and three-quarters (5,242; 1.3%; Figure 1).

The fragments concerned 132 different active substances, and 50% of them were psycholeptics or psychoanaleptics (Figure 1). The most often split tablets were preparations with pipamperone (15.8%), levodopa/decarboxylase inhibitor (10.2%), and quetiapine (6.5%; Table 3). The ten most often fragmented tablets accounted for 57% of all split tablets (Table 3).

**Figure 2:**
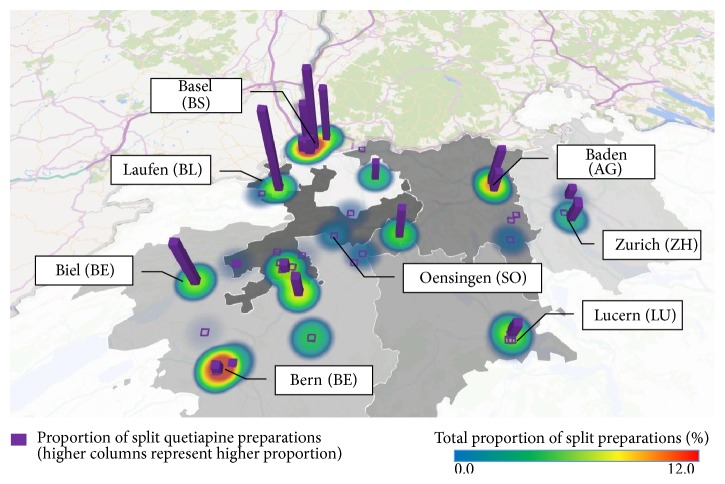
Geographical distribution of split tablets in general (heat map; the warmer the colour (i.e., red), the higher the frequency, independently of the surface) and of half quetiapine tablets (purple bars; the higher the column, the higher the proportion) for each of the 51 retirement homes (*N* = 406,956). Grey areas indicate cantonal borders. The two distant homes located in cantons SG and GR (<0.1% split tablets; no quetiapine) are not depicted.

The highest proportion of fragmented tablets was ordered for homes located in Northern Switzerland, that is, Basel (89,980; 22.1%), Bern (61,707; 15.2%), and Baden (38,503; 9.5%; Figure 2, heat map). The most split quetiapine tablets were ordered in Basel (10,273; 39%; Figure 2, bars) compared to the rest of Switzerland (i.e., French and Italian speaking parts).

## 5. Discussion

Fragments of tablets represented 8.5% of all tablets ordered in 2012 by 53 community pharmacies in Northern Switzerland for institutionalized patients. This value is probably below the effective prescription rates of fragmented tablets since splitting at the company Medifilm is reserved for cases where no lower dosage strength is available on the market. Consequently, the actual value of dispensed fragmented tablets in ambulatory setting might be higher, given that the exchange for a commercially available lower strength is not automated in community pharmacists during routine practice. A recent study in Swedish community pharmacies showed that 52.5% of the patients with a prescription for split tablets preferred whole tablets of the appropriate strength rather than split tablets [26]. Nevertheless, prescribing fragments of tablets appears to be a very common practice in the ambulatory setting.

Out of the 10 most often ordered split tablets, two (quetiapine and risperidone) had doubtful legitimacy to be fragmented since the decision cannot be backed up with the product information. Although splitting a tablet that is not intended to be fragmented does not seem to be a prescribing error [27–29], it may reduce drug effectiveness and induce toxicity and thus represents a safety issue.

Wrong prescription of 1/2 tablets usually does not cause significant patient harm, since, for many drugs, especially those with a wide therapeutic range and a long half-life, dose fluctuations are unlikely to be clinically significant. The above applies for quetiapine even more since its formulation is without functional coating or modified release.

In any case, some pitfalls exist when fragmenting tablets that are not intended to. First, patients may be easily confused about the correct dose. An effective instruction of the patients by the health professional is a prerequisite to minimise intake errors, especially when patients received information at the time of hospital discharge that diverges from the finally dispensed medication, for example, obtaining half tablets during hospitalization, leading to an initial prescription of a half tablet that is modified to one tablet of a lower dose. In the worst case, patients may split the wrong medication and take too few or too much medication. Second, patients might have poor visual acuity or dexterity that renders fragmenting very uncertain. They need at least the right tools and should be given a pill-splitting device to improve accuracy. Third, patients may store the remaining fragments or crumbles inadequately, which may affect medication stability, or use a container with no labelling, which renders a later identification of the fragments almost impossible. Fourth, patients may split several medications, which seems to be a frequent situation with 43.7% of our patients obtaining two fragments or more. Because the identification of the fragments is limited, the presence of multiple fragments represents probably the most risky situation, with a wrong intake resulting invariably from one handling error.

Given the potential risks, it is striking that half of the splitting concerned psychoactive substances in this elderly population. However, the appropriateness of splitting tablets may result from clinical observation. Because most manufacturer-based researches exclude frail elderly, and as such the appropriate dose for such patients, the prescription of split tablets may be the result of oversedation observed with whole tablets.

All above mentioned processes may represent safety issues, be time-consuming for patients, their relatives, or carers in charge of the medication managements, and ultimately generate costs that may clear the savings initially advocated for splitting tablets [30]. Finally, since handwritten prescriptions are still common, misreading by the pharmacist of one-half (1/2) as one to two (1-2) tablets can only be ruled out if prescribers would order strength and dose of the medication in milligrams [31].

The USB is a 671-bed teaching hospital in Northwestern Switzerland and serves as a major referral centre for the 1-million region. At the USB, quetiapine is administered off-label for the prevention of delirium in the postoperative setting, starting at doses of 5 mg/day with 5 mg capsules exclusively produced at the hospital pharmacy. Quetiapine is also used off-label for the therapy of delirium according to an internal scheme [32], where multiple doses of 12.5 mg up to 50 mg/24 h (<80 years) or 5 mg up to 25 mg/24 h (>80 years) are administered on the first day, with doubling of the dose on the second day. According to this scheme, therapy should be reduced or stopped after 5 days. On the wards at the USB, a dose of 12.5 mg quetiapine is administered as 1/2 tablet of 25 mg strength according to a recommendation note of the division of acute geriatrics. Quetiapine is the favourite drug for hospitalised elderly who are slightly disorientated and mildly agitated, for example, who stand up and are at risks of falling. Further, quetiapine has a short half-life, an antihistaminic action, and a lower incidence of QTc prolongation compared with haloperidol, the standard delirium therapy.

From a clinical point of view, trials on pharmacological prevention of delirium did not show conclusive results [33]. No controlled maintenance treatment trials have been conducted with quetiapine, unlike all other atypical antipsychotics which have demonstrated a positive effect on relapse prevention [15]. In studies that investigated effects on negative symptoms (emotional and social withdrawal, poverty of speech, lack of drive and motivation, and disinterest) and used haloperidol as the comparator drug, quetiapine did not show any advantage [15]. Independently of the (non)existing evidence, the internal scheme used at the USB recommends reducing or stopping treatment with quetiapine after 5 days; this information seems to get lost during hospitalisation. Neglecting annotating the duration of use, that is, the “stop” date of a treatment, represents a prescription error which may be costly [34]. Further, preventive pharmacological therapy in geriatric patients can expose them to the unnecessary risk of adverse effects. Furthermore, all antipsychotics including quetiapine are listed in the Beers Criteria as potentially inappropriate for use in elderly patients (quetiapine is an exception for patients with Parkinson's disease) [35]. Thus, continued antipsychotic therapy in geriatric patients should be reevaluated at each care transition and stopped in absence of clear indication. Particularly noteworthy is the fact that low-dose quetiapine does not seem to be administered for its antipsychotic effects but rather for its sedative effects in the elderly hospitalised patients in an empiric manner and in absence of clear evidence for a proven alternative.

Finally, it seems that the irrational case of 1/2 quetiapine 25 mg remains confined to Basel and its clinics and did not spread out. However, the level is surprisingly high when one considers that 5 years had passed since the official introduction of the recommendation in the division of acute geriatrics.

The observation that community pharmacies ordering unit-of-use soft pouch blisters were massively located in Northern Switzerland (with one marginal exception in Grisons) may reflect a cultural difference between German speaking regions in the North and French and Italian speaking regions in the South and is not a limitation.

## 6. Conclusions

Tablet splitting has a major role in dosage adjustment and should be limited to specific clinical situation, that is, titration of dose and pediatric and geriatric patients, and according to the recommendation of the product manufacturer. Physicians who prescribe a split tablet that is not intended to be fragmented and pharmacists who dispense the drug accordingly should be aware that this renders the medication unlicensed. Since resolving the uncertainty about the prescription by the pharmacists or the nurses results in much unnecessary work, splitting tablet is not suited as a method of general cost reduction. Taking into account all problems linked to the handling of a half tablet (patients' dexterity and eyesight, conservation and confusion of the halves, wastage, and therapeutic compliance), prescribing 1/2 tablet represents a safety issue. Thus, prescribers should make effort to use commercially available whole tablets. If splitting tablets is still necessary, patient counseling is recommended and pharmacies should deliver the appropriate tools or pharmacists split the tablets for the patient and repackage them.

Quetiapine 25 mg remains the third most often prescribed half tablet in Northern Switzerland in general and the first specifically in Basel. As off-label prescribing is claimed to be not evidence-based, to undermine the regulatory system, to be costly, to put the patient at risk, and to impact negatively on pharmaceutical innovation [36], this situation is more than frightening. It is usually in the company's interests to extend the indications of its products. However, in this particular case, the pharmaceutical industry seems to limit its investment probably because generic formulations are available. Pharmaceutical companies should be encouraged to introduce new strengths to an existing range of products, in view of an optimisation of seamless care between the different health care professionals.

## Figures and Tables

**Table 1:** Fragments and half quetiapine tablets by home (*N* = 53) as frequency and as proportion of the total number of fragments (*N* = 406,956). The cantons are given by their official abbreviations: BE, Bern: BS, Basel-Stadt; AG, Aargau; SO, Solothurn; BL, Basel-Landschaft; LU, Lucerne; ZH, Zurich; SG, St. Gallen; GR, Grisons.

Home ID	Number of fragments (%)	Number of half quetiapine tablets (%)	Cantons								
			BE	BS	AG	SO	BL	LU	ZH	SG	GR
1	48300 (11.9)	409 (0.1)	x								
2	28255 (6.9)	1142 (0.3)	x								
3	22835 (5.6)	2445 (0.6)	x								
4	22415 (5.5)	578 (0.1)		x							
5	21996 (5.4)	988 (0.2)			x						
6	21178 (5.2)	3699 (0.9)		x							
7	20853 (5.1)	2478 (0.6)		x							
8	20840 (5.1)	458 (0.1)				x					
9	19557 (4.8)	3862 (0.9)					x				
10	18992 (4.7)	1597 (0.4)			x						
11	16065 (3.9)	778 (0.2)						x			
12	15756 (3.9)	1774 (0.4)			x						
13	14568 (3.6)		x								
14	14517 (3.6)	862 (0.2)					x				
15	13368 (3.3)	21 (0.01)	x								
16	11539 (2.8)	854 (0.2)							x		
17	10083 (2.5)	2133 (0.5)		x							
18	9861 (2.4)	582 (0.1)		x							
19	5963 (1.5)							x			
20	5868 (1.4)					x					
21	4990 (1.2)		x								
22	4968 (1.2)				x						
23	4466 (1.1)						x				
24	3518 (0.9)	775 (0.2)		x							
25	3124 (0.8)						x				
26	2526 (0.6)	523 (0.1)							x		
27	2480 (0.6)					x					
28	2435 (0.6)					x					
29	2334 (0.6)									x	
30	1456 (0.4)					x					
31	1427 (0.4)						x				
32	1078 (0.3)			x							
33	1004 (0.2)		x								
34	980 (0.2)	28 (0.01)		x							
35	905 (0.2)					x					
36	890 (0.2)					x					
37	836 (0.2)	126 (0.03)							x		
38	771 (0.2)					x					
39	751 (0.2)	223 (0.1)			x						
40	719 (0.2)							x			
41	514 (0.1)							x			
42	414 (0.1)					x					
43	291 (0.1)					x					
44	276 (0.1)							x			
45	267 (0.1)				x						
46	224 (0.1)				x						
47	159 (<0.1)				x						
48	133 (<0.1)					x					
49	125 (<0.1)								x		
50	39 (<0.1)		x								
51	19 (<0.1)										x
52	14 (<0.1)			x							
53	14 (<0.1)		x								
Total	**406,956 (100%)**	**26,356 (6.5%)**	**8**	**9**	**8**	**11**	**5**	**5**	**3**	**1**	**1**

**Table 2:** Number of split medications by patient (*N* = 1,321 patients).

Number of fragments	Number of patients (%)	Cumulative number of patients (%)
1	744 (56.3)	744 (56.3)
2	350 (26.5)	1,094 (82.8)
3	139 (10.5)	1,233 (93.3)
4	65 (4.9)	1,298 (98.2)
5	15 (1.1)	1,313 (99.3)
6	5 (0.4)	1,318 (99.7)
7	2 (0.2)	1,320 (99.9)
8	1 (0.1)	1,321 (100)

**Table 3:** Ten most frequently split medications given by active substances (SPC: Summary of Product Characteristics).

Active substance (original brand name)	Proportion of split tablets [%]				Splitting is explicitly mentioned in the SPC (yes/no)
	Total (cumulative)	Quarter 1/4	Half 1/2	Three-quarter 3/4	
Pipamperone (Dipiperon)	15.8	6.2	9.3	0.3	y
Levodopa/decarboxylase inhibitor (Madopar)	10.2 (26.0)	—	10.1	0.1	y
Quetiapine (Seroquel, Sequase)	6.5 (32.5)	0.3	6.2	—	n
Lorazepam (Temesta)	5.1 (37.6)	0.4	4.7	—	y
Mirtazapine (Remeron, generics)	4.3 (41.9)	—	4.3	—	y
Torasemide (Torem, generics)	3.9 (45.8)	2.2	1.2	0.5	y
Zolpidem (Stilnox, generics)	3.2 (49.0)	—	3.2	—	y
Metoprolol (Beloc ZOK, generics)	2.7 (51.7)	—	2.7	—	y
Citalopram (Seropram, generics)	2.7 (54.4)	—	2.7	—	y
Risperidone (Risperdal)	2.6 (57.0)	—	2.6	—	n
